# Rectal adenocarcinoma in patients with anorectal malformations: report of two cases and a review of the literature

**DOI:** 10.1186/s40064-016-3263-5

**Published:** 2016-09-20

**Authors:** P. Midrio, S. Battaglia, E. Urso, M. Castagnetti, P. Gamba

**Affiliations:** 1 Department of Mother and Child, Ca’ Foncello Hospital, 31100 Treviso, Italy; 2 Department of Woman and Child Health, University Hospital of Padova, Padua, Italy; 3 Department of Surgical Gastroenterologic and Oncologic Science, University Hospital of Padova, Padua, Italy; 4 Urology Unit, Section of Paediatric Urology, University Hospital of Padova, Padua, Italy

**Keywords:** Anorectal malformation, Rectal cancer, Urethral fistula, Pull-through, Recto-vestibular fistula

## Abstract

**Aim:**

Anorectal malformation (ARM) is a rare congenital disorder of the anus and rectum. In the last 30 years virtually all patients born with ARM have survived and surgeons from adult care may be called to deal with new and long-term *sequelae*, including tumors of the pulled-through anorectum. Two new cases of colorectal carcinoma in young adults born with ARM and a review of the literature is reported to emphasize the importance of a multidisciplinary follow-up.

**Methods:**

A man and a woman, with previous history of ARM, presented at 34 years of age with symptoms of intestinal occlusion and a large pelvic mass. Both patients had no familial history of colorectal carcinoma.

**Results:**

The patients underwent biopsies (mucinous rectal adenocarcinoma) and stadiation (T4N0M0). In one case the microsatellite instability showed a stable profile. Despite maximal treatments, including surgery, chemo- and radio-therapy, they both died a few years after diagnosis for progression of disease.

**Conclusion:**

Case studies are too limited to suggest guidelines for prevention and treatment of such complications, but the life-long follow-up is mandatory in the framework of a well-established network between pediatric and adult surgeons. The risk of tumor development in these patients should not be neglected and colleagues from adult care should be aware of the possibility this occurs in their practice.

## Background

Anorectal malformation (ARM) is a rare congenital disorder of the anal canal, rectum, and colon occurring in 1.33 to 4.93 per 10,000 births worldwide (The Centre for International Clearinghouse for Birth Defects Surveillance and Research 2012). Associated anomalies can concern nearby organs, such as urogenital tract and spinal cord, as well as distant ones, such as oesophagus, heart, vertebral column, and skeleton (Solomon 2011). The abdomino-perineal pull-through procedure was the standard treatment for ARM until 1982 when de Vries and Peña described the posterior sagittal anorectoplasty (Levitt and Peña 2012). Known complications of surgery for ARM include faecal incontinence, soiling, constipation, and rectal stricture (Levitt and Peña 2012).

In the last 30 years, virtually all patients with ARM without associated life-threatening conditions have survived. For this reason, physicians now face the problem to deal with new and long-term *sequelae* of the correction of an ARM. Urinary tract deterioration and sexual dysfunctions have emerged as major clinical problems that can often affect these patients long-term (Giuliani et al. 2013; Grano et al. 2008). In the same way, cases of malignant degeneration of the pulled-through anorectum have occasionally been reported (Mukawa et al. 1988; Polk et al. 1982; Posey et al. 2000; Ou et al. 2007; Symons et al. 2010; Ahmed et al. 2012; Clark et al. 2002; Gupta et al. 2012; Violi et al. 2001).

Colorectal carcinoma (CRC) represents the third most common cancer in men, with an incidence of 22 cases per 100,000/year, and the second in women, with an incidence of 15 cases per 100,000/year worldwide (Ferlay et al. 2013). Its frequency peaks at age 65 years (Hill et al. 2007).

We report two patients with ARM corrected at birth who developed CRC early in life. These cases, together with a review of those previously reported, emphasize the importance of life-long follow-up in patients born with ARM.

## Case 1

A 34 year-old woman presented at another hospital with abdominal pain, fever and episodes of rectal bleeding. The patient had no family history of cancer. She was born with a recto-vestibular fistula that had been corrected at 3 months of life using the pull-through technique. Due to persistent severe fecal incontinence, she required bowel management with occasional use of glycerine and had undergone multiple continence surgeries including sphincter-restoring surgery by right anograciloplasty at 9 years of age and subsequent implantation of an anal electrostimulator (implantable pulse generator) at 27 years of age. Moreover, at 29 years of age, the patient had undergone open left adnexectomy for an ovarian cyst, which turned out to be an endometrial cyst.

At presentation, pelvic US and abdominal CT showed the presence of a pelvic mass, that was considered as a relapse of endometriosis. Recto-sigmoidoscopy demonstrated, at 8 cm from anal verge, an ulcerated rectal mass of 4 cm in diameter, whose biopsies, however, were negative for malignancy. Via a laparotomy, 6 cm mass between rectum and uterus, corresponding to the ulcerated mass, adherent also to the presacral plain and vagina, was biopsied, and a diverting left colostomy created. At histology, a well-differentiated mucinous adenocarcinoma was diagnosed without evidence of tumor in the surrounding tissues. No metastases were detected at staging imaging. Therefore, the patient was staged as pT4 pN0 pM0 and started on neo-adjuvant chemotherapy with Folfox [oxaliplatin, fluorouracil (5FU) and folic acid] for 9 months. In retrospect, the paraffin embedded specimens of the tumor, reviewed years later for this study, were not adequate for proper DNA extraction and microsatellite instability test was not possible.

Three months after chemotherapy, the patient was re-admitted for intestinal occlusion. At CT scan a mass of 13 × 10 cm was detected. Resection of rectum, uterus, right ovary, vagina, vulva, and part of bladder and left ureter were performed, and a left ureteroneocistostomy performed. The post-operative course was complicated by perineal wound dehiscence, that required removal of the anograciloplasty and anal electro stimulator, and, later, by development of an urethral fistula that required placement of bilateral nephrostomies. The adjuvant therapy was not completed due to a series of septic events and 6 months later lung and para-aortic metastasis were detected. Chemotherapy with fluorouracil (5FU) and avastin was attempted until patient demise at age 36 years for disease progression.

## Case 2

A 34-year old man presented to another hospital with persistent discharge of brownish gelatinous material with urine, constipation and soiling lasting from 6 months. The patient, without family history of cancer, was born with a recto-prostatic fistula and underwent staged repair of ARM. Because of extended colic resection and the anorectal malformation the patient never reached a satisfactory fecal continence and was on a bowel management program including daily enemas of 300 cc of water and 100 cc of glycerin.

**Fig. 1:**
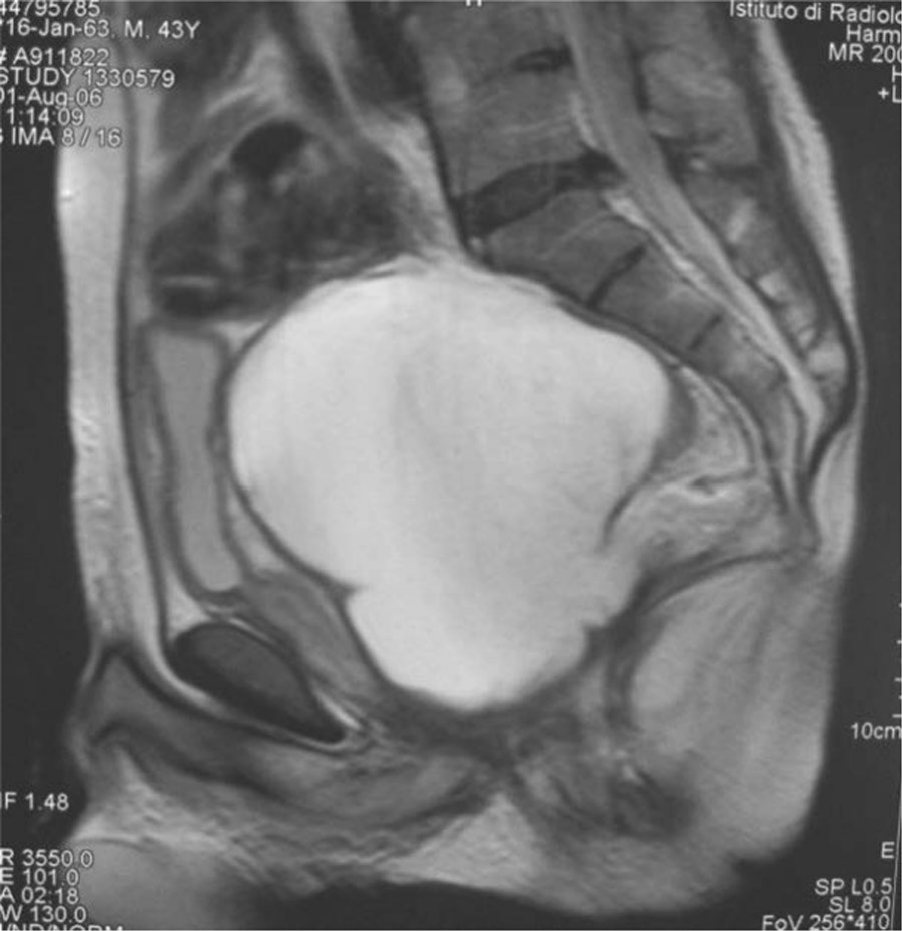
Magnetic resonance of the pelvis shows a lobulated, well-limited liquid mass 12 cm in diameter, in contact with prostatic urethra and compressing bladder and rectum

**Fig. 2:**
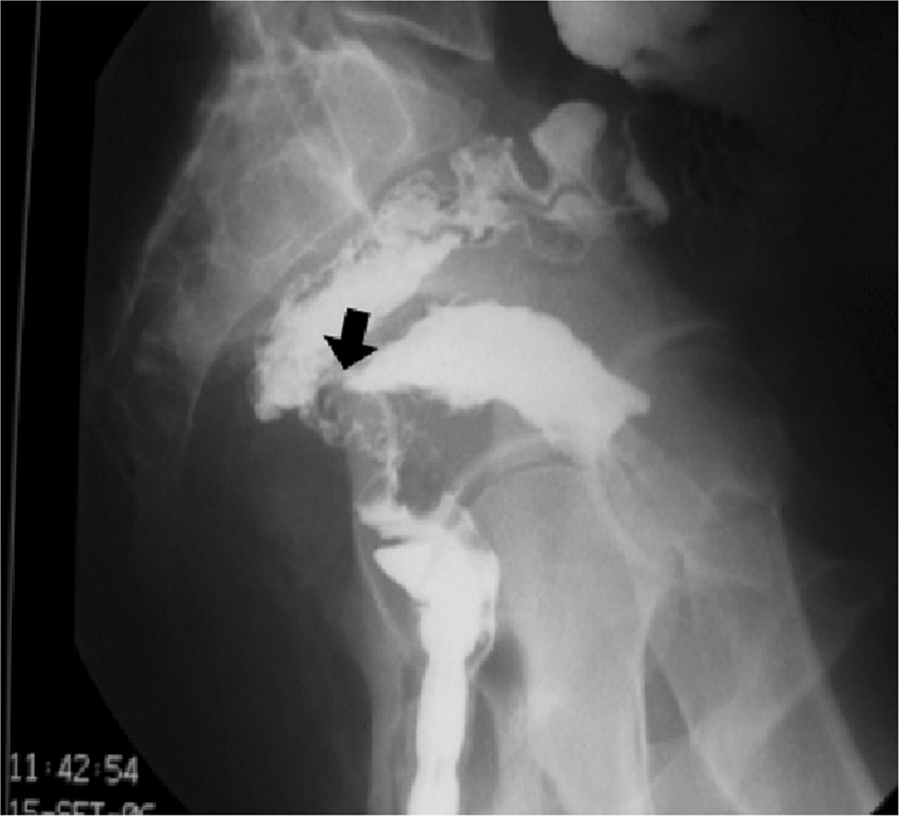
Contrast enema demonstrates a communication (*arrow*) between the pelvic mass and the rectum

At rectal examination, a mass was palpable and an US revealed a 10 cm pelvic mass behind the bladder. At MRI, a huge cystic mass between the prostatic urethra and rectum was detected and the patient was referred to our centre (Fig. 1). Because of prevalence of intestinal signs and symptoms, a rectoscopy was initially performed. It revealed compression and deviation of rectum that presented with fragile and bloody mucosa, interpreted as an accumulation of glycerine in a sort of rectal pseudodiverticulum. The pelvic collection, 400 cc of gelatinous material, was drained thru the rectum with rapid restoration of bowel and urinary functions. Pathology of fluid revealed amorphous eosinophilic material. A contrast enema showed a communication between the rectum and the residual pelvic mass (Fig. 2). A few months after the drainage, a pelvic mass was again detected on US, associated with sub-occlusive symptoms and monthly urinary tract infections. A “faecal-fluid collection” was again drained and was deemed not suggestive of any malignancy at histology. Imaging (pelvic CTs, contrast enema, urethro-cistography, and urethro-cistoscopy) failed to show any recto-urethral fistula. Because of persistence of sub-occlusive symptoms the patient underwent a temporary double barrel ileostomy at the age of 36 ½ years.

**Fig. 3:**
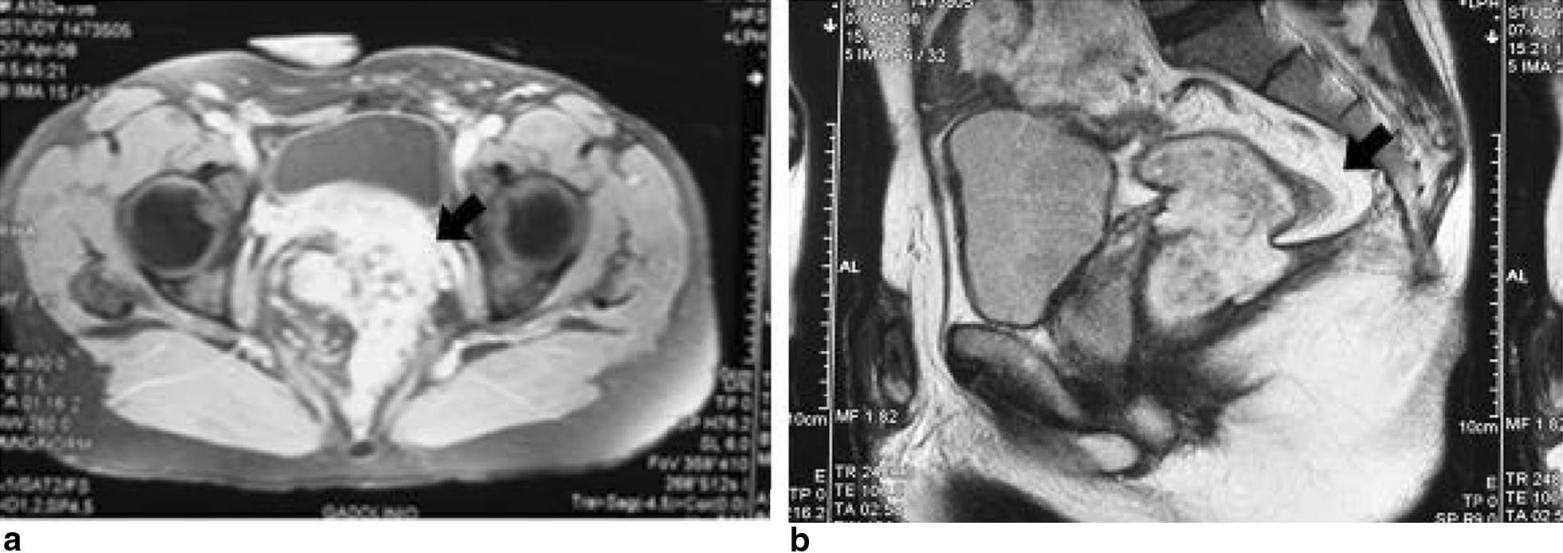
**a** MRI transverse plane **b** sagittal plane. An inhomogeneous pelvic mass of 7 × 7 × 4 cm in size, with thick and vascularised walls (*arrow*) is visible, compressing the bladder anteriorly, reaching the ano-rectal junction inferiorly and communicating with rectal lumen

One year later, as bouts of urinary tract infections occurred, another rectoscopy was performed that showed a granulomatous nodule on the anterior rectal wall. Pathology was a villous adenoma with high-grade dysplasia. An abdomino-pelvic MRI (Fig. 3a, b) revealed a new pelvic 7 × 7 × 4 cm mass communicating with the anterior rectal wall and with prevalent outgrowing aspects. New endoscopic rectal biopsies of the intraluminal part of the lesion, originating at 8 cm from the anal verge, revealed a well differentiated colonic adenocarcinoma.

The patient (now 37 years old) underwent Hartmann operation and closure of previous ileostomy. At surgery the mass appeared mainly extraluminal, contained in a sort of thin rectal wall pseudodiverticulum and, at pathology, resulted a mucinous adenocarcinoma of large intestine pT4 pN0 pM0. All biopsies of resection margins were negative for malignancy. Microsatellite instability was investigated using standard Bethesda microsatellites panel (Umar et al. 2004), resulting in a stable profile. Adjuvant 5FU based chemotherapy and 50.4 Gys radiotherapy were completed.

Despite treatment, local recurrence was detected in the rectal stump 8 months after surgery and perineal resection of anus and spared rectum was required. At histology, a moderately differentiated adenocarcinoma of large intestine with mucinous component, infiltrating the bladder wall, sacrum and pelvic floor. The patient received, then, an intensified cycles chemotherapy (Folfox treatment).

Nonetheless, 21 months after last intervention, perineal neoplastic ulcer and lung metastases occurred and the patient died at 41 years of age for progression of disease.

## Discussion

We report two cases of rectal adenocarcinoma aroused in young adulthood in patients born with anorectal malformation. The patients, a male and a female, were both 34 years old at onset of symptoms and none of them had family history of CRC. Both presented with a large pelvic mass, that resulted to be a mucinous rectal adenocarcinoma, and were treated with surgical and chemo-radio therapy but they died for progression of disease a few years after diagnosis. Because of the young age at diagnosis and tumor histotype, the mismatch repair deficiency was investigated by mean of microsatellite instability, resulting in a non-informative test (case 1) and in a negative test (case 2).

Adenocarcinoma of the rectum rarely occurs in patients younger than 30 years; at this age, it represents 2.1 % of all malignancies (Spunt et al. 2006). Meyer et al. (2010) studied the incidence of CRC in a population aged <40 years and they found it was 1.11 per 100,000 for the colon cancer and 0.42 per 100,000 for the rectal cancer.

Mucinous adenocarcinoma is a rare morphological subtype of colorectal cancer characterized by abundance of extracellular mucine (≥50 % of the volume) secreted by overactive neoplastic acinar cells (Chand et al. 2014) and represents from 5 to 15–20 % of all colorectal cancer (Chand et al. 2014; Hyngstrom et al. 2012). The incidence of this histological subtype is higher in younger patients, therefore at present times it is difficult to demonstrate ARM may have played a role in the etiopathogenesis of the disease in these two patients, as the incidence of this tumor in the ARM population compared to the general population is not known. Depending on different studies, rectal mucinous cancers are found to be more common below the age of 50 or even below the age of 39 (Chand et al. 2014; Wu et al. 1996). Most mucinous tumors are found in the right colon (60 % of all mucinous tumors) and only 18 % in the rectum (Hyngstrom et al. 2012). With regard to clinical outcome and survival, mucinous cancer of colon and rectum show a worse prognosis if compared with non-mucinous types (Chand et al. 2014; Hyngstrom et al. 2012). This is true especially for rectal cancers, which are independently associated with a higher risk of death when compared to non-mucinous adenocarcinomas (Hyngstrom et al. 2012).

Predisposing factors for colorectal carcinoma in children and young adults include hereditary conditions (Hereditary Non Polyposis Colorectal Cancer Syndrome, HNPCC) (Umar et al. 2004), inflammatory bowel disease, previous radiation exposure. Ureterosigmoidostomy (USM) is a well-known postoperative pediatric condition at increased risk of colorectal cancer when patients operated for bladder exstrophy reached the adult age (Khan et al. 2004). It is reported, indeed, that about 5 % of patients undergoing USM will develop colon cancer (Spunt et al. 2006), with an increased risk of colorectal cancer by 100 fold if compared to general population (Gupta et al. 2012).

Malignancies of a pulled-through anorectum are extremely rare, being reported in only ten patients (Mukawa et al. 1988; Polk et al. 1982; Posey et al. 2000; Ou et al. 2007; Symons et al. 2010; Ahmed et al. 2012; Clark et al. 2002; Gupta et al. 2012; Violi et al. 2001). Nine of these cases were males; only three of them were younger than 40 years of age, and in just one patient the mucinous carcinoma was detected (Symons et al. 2010). In three cases the authors postulated that a possible cause of carcinogenesis was the presence of a recurrent or misdiagnosed communication between urinary and intestinal tract. Table 1 summarizes data about patients reported in literature, including our owns.

**Table 1:** Summary of reported cases of carcinoma in patients with imperforate anus [Adapted from (Ou et al. 2007)]

Case	Age/sex	Type of anorectal malformation	Concomitant GU anomaly	Location of carcinoma	Type of carcinoma	Reference
1	63/M	Rectourethral fistula (bulbar)	Hypospadia	Rectosigmoid colon (12 cm from anal verge)	Adenocarcinoma	Polk et al. (1982)
2	35/M	Rectourethral fistula (prostatic)	None	Anorectum	Adenocarcinoma	Mukawa et al. (1988)
3	25/M	Not mentioned	Hypospadia Neurogenic bladder	Rectosigmoid colon (2 cm from anal verge)	Mucinous adenocarcinoma	Posey et al. (2000)
4	21/M	Rectoperineal fistula	None	Anorectum	Adenocarcinoma (poorly differentiated)	Ou et al. (2007)
5	44/M	Not mentioned	Not mentioned	Retrorectal	Mucinous adenocarcinoma	Symons et al. (2010)
6	53/M	Rectourethral fistula	Not mentioned	Pararectal	Mucinous adenocarcinoma	Symons et al. (2010)
7	40/M	Not mentioned	Not mentioned	Neorectum	Adenocarcinoma	Ahmed et al. (2012)
8	43/M	Recurrent misdiagnosed rectourethral fistula	Not mentioned	Neorectum (2 cm from anal verge)	Adenocarcinoma	Gupta et al. (2012)
9	65/F	Rectovestibular fistula (never treated)	Left renalhypoplasia	Rectum (7 cm from anal verge)	Adenocarcinoma	Violi et al. (2001)
10	60/M	Colovesical fistula (never treated)	Right crossed fused renal ectopia	Splenic flessure	Adenocarcinoma	Clark et al. (2002)
Author’s 1	34/F	Rectovestibular fistula	None	Anorectum (8 cm from anal verge)	Mucinous adenocarcinoma	
Author’s 2	34/M	Rectourethral fistula (prostatic)	None	Anorectum (8 cm from anal verge)	Mucinous adenocarcinoma	

From the analysis of literature, the theory suggested by Symons et al. (2010) that neoplasms originate from remnants of rectal mucosa outside neo-rectum after pull-through procedure and rectal invasion is the second step in neoplasm growing, could apply to both our cases and seems to be a valid hypothesis. In case 2 one of the previous mentioned cancer predisposing factors (previous recto-urinary fistula) was present and it could be postulated remnants of rectal mucosa of the original recto-urethral fistula may have been in contact with urinary stream for years, in this way triggering the well-known carcinogenetic pathway. The cancer may have subsequently grown mainly outside the urinary stream becoming detectable at colonoscopy, thus justifying the initial brief urinary signs and late persistent abdominal signs and symptoms of case 2. The last assumption could also explain the great prevalence of adenocarcinoma in males treated for anorectal malformations reported in literature. Regarding females, the surgical techniques to correct a recto-vestibular fistula are quite different and remnants of rectal mucosa may be left outside the rectum, even in continuity with the vaginal wall, thus explaining the occurrence of rectal cancers in such anorectal malformations.

In both our patients, the diagnosis was established using rectoscopy or recto-sigmoidoscopy and we believe the rectal origin of such masses should be the first working hypothesis in patients with a surgical history of a rectal pull-through for anorectal malformations. Nevertheless, another rare condition that may have a similar presentation is a posterior urethral diverticulum imprinting the rectum. In adults, posterior urethral diverticula are usually acquired and occur in patients with a history of urethral strictures and multiple urethrotomies (Guneri et al. 2016). However, posterior urethral diverticula can also be a specific complication of the repair of anorectal malformations with recto-urethral fistulas (Pandey et al. 2014; Takazawa et al. 2014). Therefore, if the recto-sigmoid endoscopy is not conclusive, a retrograde and voiding cystourethrography plus a urethroscopy should be considered to complete the work-up.

These speculations would benefit from more consistent data but case studies are still very limited to give guidelines for the prevention and treatment of such complications. However, we believe that life-long follow-up in such patients is mandatory and it should also include investigations for early diagnosis of malignancies. For that purpose, we agree with Ou et al. (2007) who suggest life-long annual follow-up with manual rectal examination for patients operated for imperforate anus. Moreover, Woodhouse (2002) proposes flexible sigmoidoscopy once a year for patients with USM, beginning 10 years after surgery. This procedure could be proposed to patients with ARM, starting in young adulthood, as cases of adenocarcinoma are described in 25–30 years old patients. Finally, the MRI has the ability to differentiate mucinous tumors from non-mucinous ones and it should be included in the pre-operative plan (Chand et al. 2014).

If patients treated in their infancy for ARM will develop colorectal cancer in their young adulthood or later, it is of paramount importance the creation of a strong and well established network between pediatric and general surgeons. The first ones must report patients to the second ones, explaining history and possible complications, while general surgeon must be aware of diagnostic technique and treatment of such complications.

In conclusion, ARM are rare congenital anomalies and rectal adenocarcinoma arousing in those patients is extremely rare, but life span is expected to be normal for this kind of patients and diagnosis and therapy are still challenging. We believe that nowadays the management of ARM patients must be shared within an expert and trained team including several specialties (pediatric and adult surgery, urology, pediatrics, gynecology, psychology) and a preventive program for colorectal cancer. The risk of tumor development in these patients should not be neglected and colleagues from adult care should be aware of the possibility this occurs in their practice.
